# Systematic evaluation of underlying defects in DNA repair as an approach to case-only assessment of familial prostate cancer

**DOI:** 10.18632/oncotarget.5554

**Published:** 2015-10-14

**Authors:** Emmanuelle Nicolas, Sanjeevani Arora, Yan Zhou, Ilya G. Serebriiskii, Mark D. Andrake, Elizabeth D. Handorf, Dale L. Bodian, Joseph G. Vockley, Roland L. Dunbrack, Eric A. Ross, Brian L. Egleston, Michael J. Hall, Erica A. Golemis, Veda N. Giri, Mary B. Daly

**Affiliations:** ^1^ Programs in Genomics, Fox Chase Cancer Center, Philadelphia, PA, USA; ^2^ Programs in Molecular Therapeutics Fox Chase Cancer Center, Philadelphia, PA, USA; ^3^ Programs in Biostatistics, Fox Chase Cancer Center, Philadelphia, PA, USA; ^4^ Kazan Federal University, Kazan, Russia; ^5^ Inova Translational Medicine Institute, Inova Health System, Falls Church, VA, USA; ^6^ Cancer Prevention and Control, Fox Chase Cancer Center, Philadelphia, PA, USA; ^7^ Sidney Kimmel Cancer Center at Thomas Jefferson University, Philadelphia, PA, USA

**Keywords:** familial prostate cancer, whole exome sequencing, DNA damage response, genetic susceptibility to prostate cancer, case-only study

## Abstract

Risk assessment for prostate cancer is challenging due to its genetic heterogeneity. In this study, our goal was to develop an operational framework to select and evaluate gene variants that may contribute to familial prostate cancer risk. Drawing on orthogonal sources, we developed a candidate list of genes relevant to prostate cancer, then analyzed germline exomes from 12 case-only prostate cancer patients from high-risk families to identify patterns of protein-damaging gene variants. We described an average of 5 potentially disruptive variants in each individual and annotated them in the context of public databases representing human variation. Novel damaging variants were found in several genes of relevance to prostate cancer. Almost all patients had variants associated with defects in DNA damage response. Many also had variants linked to androgen signaling. Treatment of primary T-lymphocytes from these prostate cancer patients versus controls with DNA damaging agents showed elevated levels of the DNA double strand break (DSB) marker γH2AX (*p* < 0.05), supporting the idea of an underlying defect in DNA repair. This work suggests the value of focusing on underlying defects in DNA damage in familial prostate cancer risk assessment and demonstrates an operational framework for exome sequencing in case-only prostate cancer genetic evaluation.

## INTRODUCTION

Prostate cancer (PCa) is the most common noncutaneous cancer and the second-leading cause of cancer-related death in men in the United States [1]. Prior studies have shown that family history, such as a brother or father with PCa or relatives affected at an early age, is a major risk factor [2, 3]. A growing consensus in the field is that inherited factors for PCa are heterogeneous, involving gene mutations of high penetrance that occur in a small number of families, but also low or moderately penetrant alterations that are more common, and which may interact in individuals to promote disease [4–7]. While a few genes such as *BRCA2* and *HOXB13* are definitively linked to prostate cancer risk in specific patient populations [8–10], a greater proportion of prostate cancer risk may be associated with common alleles of low-to-intermediate penetrance or private alleles in families contributing to cancer risk. While panel testing may be useful in detecting some of these variants, it is difficult to design a panel that adequately captures the rapidly increasingly number of rare variants associated with multiple forms of cancer. With the cost of DNA sequencing rapidly decreasing, analysis of exome and genome data is becoming an alternative approach. However, given the computational complexity of assessing the many rare variants found in every individual [11, 12], particularly if multiple independent variants may be interacting to produce risk [13], it is important to employ a robust analytic pathway grounded in understanding of the physiological basis of the disease.

In addition to genetic heterogeneity of prostate cancer susceptibility, another common scenario in clinical cancer risk evaluation that impacts the assessment of genetic variants is a “case-only” presentation for genetic testing [14]. This may arise when a patient presents for cancer risk evaluation with small family structure, limited family history information, and limited access to specimens from other affected relatives due to death or other causes. In this situation, the ability to clarify cancer susceptibility of genetic variants using family history or by testing a DNA sample from one or more informative blood relatives (affected or unaffected with cancer) is not possible. Such prostate cancer pedigrees will often be characterized by other cancers, raising the possibility that some inherited variants may be risk factors for multiple cancer types. In the clinical cancer risk assessment setting, novel pathway-based approaches to identifying at-risk individuals and families are greatly needed.

In this study, we have tested the hypothesis that germline exome data from individuals with prostate cancer and a family history of one or more cancer types would be enriched in damaging variants falling into two classes. The first class includes variants in genes associated with defects in the DNA damage response (DDR) pathway, reflecting the growing recognition that a number of genes implicated in PCa risk, such as *BRIP1, MSH2, MSH3, CHEK2*, and *PALB2*, have general function in contributing to early genomic instability in multiple cancers [15–19]. Of particular relevance to prostate cancer, the androgen receptor has been found to regulate a suite of DDR genes, including some that promote resistance to radiotherapy in prostate cancer in part by promotion of non-homologous end joining (NHEJ) repair [20]. The related second class of variants includes those in genes of sex hormone metabolism previously implicated as relevant to the pathogenesis of non-familial prostate cancer [21], including those that regulate androgen signaling by various mechanisms. Here we present data on specific pathways analysis of exomes of a group of case-only prostate cancer patients who underwent clinical genetic evaluation for inherited cancer risk based on personal and/or family cancer features. We identify likely predisposing variants in every case, with a particular bias towards evidence of DDR-impairing defects in most cases. Based on this work, we perform functional testing that directly demonstrated increased sensitivity to DNA damaging agents in lymphocytes from prostate cancer patients bearing predicted DNA damaging alleles. This work begins to develop an operational framework for exome sequencing in case-only prostate cancer genetic evaluation.

## RESULTS

### Development of a high value list of candidate genes

To meet the goal of developing an operational framework for assessing case-only patients, we developed a comprehensive, hypothesis-based candidate gene list for particular scrutiny (Supp. Table S1). To this end, we integrated top scoring candidates from a number of existing sources that collated genes based on orthogonal selection criteria (Table 2). The primary hypothesis for this purpose was that rare variants leading to defects in DDR would be important in predisposition for general cancer risk, while the secondary hypothesis was that rare variants damaging genes associated with androgen signaling or prostate function would provide a bias for cancer in the prostate.

**Table 2:** Sources for building the candidate gene list

Description	Source/Reference	Number of genes
DNA repair genes	Wood lab http://sciencepark.mdanderson.org/labs/wood/DNA_Repair_Genes.html	179
AR-regulated DNA repair genes	(Polkinghorn, et al., 2013)	144
AR interactors	Human protein reference database	149
BRCA1 interactors	http://www.hprd.org/	102
Genes most frequently mutated in prostate tumors	(Barbieri, et al., 2012)	19
	(Grasso, et al., 2012)	
TARGET	(Van Allen, et al., 2014)	130
Genes linked to androgen and estrogen biosynthesis and metabolism	(Sun, et al., 2011)	30
Literature mining for genes involved in prostate cancer	MalaCard prostate cancer, (Rappaport, et al., 2014) http://www.malacards.org/card/prostate_cancer	Top 50
	Diseases-Jensen lab-University of Copenhagen, (Pletscher-Frankild, et al., 2015) http://diseases.jensenlab.org	Top 150
Candidate genes list for exome study of LNCaP cell line	(Spans, et al., 2012)	Top 50
Genes linked to glycosylation disorders	(Freeze, et al., 2014)	103

In addition to sources noted in the Results section, genes were added from online resources (MalaCards and DISEASES), which use literature mining to identify genes linked to inherited and somatic prostate cancer, a study by Spans et al, describing mutations distinguishing the widely studied LNCaP prostate cancer cell line from normal prostate cells (Spans, et al., 2012), and a group of genes linked to glycosylation disorders (Freeze, et al., 2014), given the growing evidence for defects in proteins involved in glycosylation in the pathogenesis of prostate cancer (Drake, et al., 2015).

**Figure 2:**
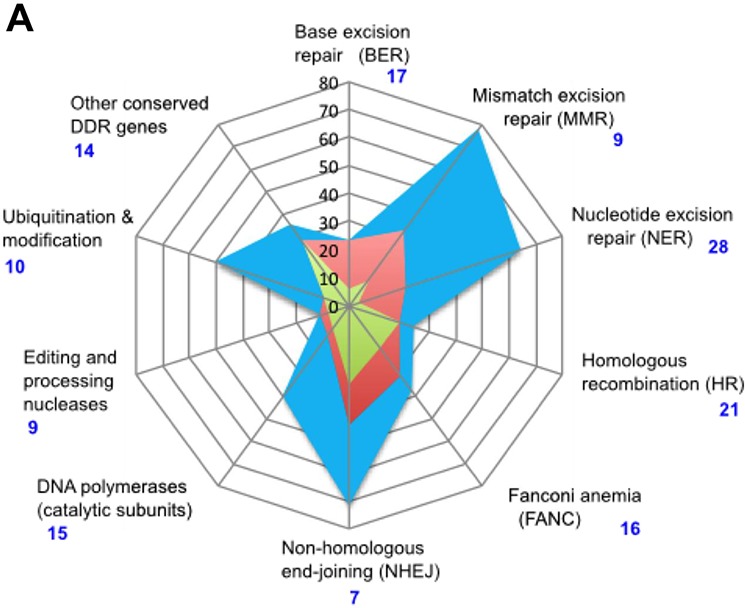

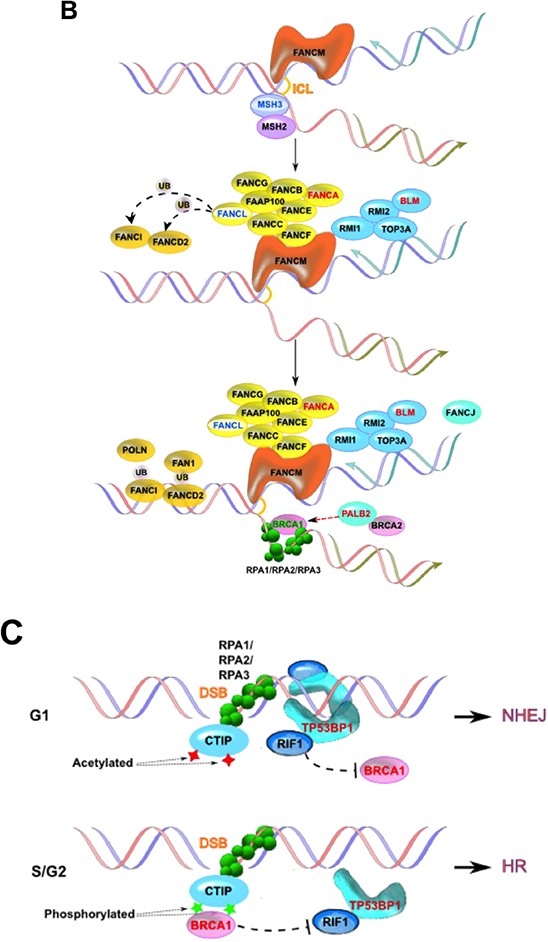
DNA damage response genes in prostate cancer patients **A.** Radar plot indicating percent of genes that are AR-associated (blue), induced by androgens (red) or direct AR targets (green) in each class of DNA repair genes, based on [20]. Classes of DDR genes are based on the list posted at the Wood lab Web site (see Table 2), except that the two classes “Base excision repair (BER)” and “Other BER and strand break joining factors” were merged. Vertical black numbering indicates percent of AR-associated genes; blue numbering indicates number of genes in each class. **B.** Simplified representation of DNA interstrand crosslink damage being repaired by proteins in Fanconi's anemia pathway [101]. Variants found in patient 124604 are indicated in red font; those found in patient 117939, in blue font.**C.** Alternative binding by TP53BP1 or BRCA1 (shown in red font) specifies NHEJ versus HR DNA repair, with variants in each gene found in patient 129413.

To identify a relevant candidate set we queried the Wood group website, which maintains an updated comprehensive list of genes linked to DNA damage response [35]. This list was extended with genes described by Polkinghorn and colleagues, who have noted a subset of DNA repair genes specifically regulated by the androgen receptor (AR) [20] (see Figure 2A). The Human Protein Reference Database (HPRD) [36] provided lists of genes that interacted physically or functionally with the well-validated risk factor BRCA1, as well as the AR. Providing broader context relevant to AR signaling, several recent studies have provided lists of genes mutated at appreciable frequencies in sporadic prostate cancer [37, 38], including castration resistant prostate cancer [39]. The TARGET database (Tumor Alterations Relevant for Genomics-Driven Therapy) [38] provides a broader list of genes of clinical value for cancer treatment, based on their roles as tumor drivers. Sun and colleagues developed a list of single nucleotide polymorphisms (SNPs) affecting sex hormone metabolism, some of which showed significant or near-significant linkage to prostate cancer aggressiveness at diagnosis [21].

Finally, as a tertiary hypothesis, we also considered that non-rare variants, or variants in genes linked to cancers other than prostate, might contribute to prostate cancer risk in some circumstances. Typically, a threshold of 1% for a minor frequency allele (MAF) is used to filter out non-rare variants as insignificant in the absence of clear clinical indication of phenotypic effect [14]. However, a growing body of evidence suggests that cancer risk reflects the interaction of multiple predisposing factors [13, 40, 41], suggesting such non-rare variants may interact with specific rare variants. We also considered in our analysis non-rare variants in genes predicted as relevant to somatic or hereditary cancers by the Ambry report.

### Identification of genetic variants in the prostate cancer patient cohort

Focusing on this candidate list, we analyzed rare single nucleotide variants (SNVs) causing missense mutations, frameshift or nonsense mutations predicted to result in early truncation of protein and short in-frame insertions or deletions (indels) (Supp Tables S2, S3). SNVs were only considered further if they passed an initial test in which multiple predictor algorithms indicated the variant would disrupt protein function (see Methods and Supp Table S2 for comprehensive analysis).

Table 3 summarizes the significant variants found in each of the 12 patients (with extended information in Supp Table S4). All variants listed in Table 3 passed Sanger validation. Each patient had variants affecting 3 to 7 genes on the candidate list. All 12 patients had reported being white and non-Hispanic: Table 3 describes the frequency of each variant in the subset of ~30,000 European non-Finnish individuals of the Exome Aggregation Consortium (ExAC) database; these frequencies were in agreement with those reported in an independent (ITMI) cohort of 634 white non-Hispanic individuals who denied a personal or family history of cancer (Supp Table S4). Thirty alterations identified in the prostate cancer patients in this study were found in fewer than 20 individuals in the ExAC database (< 0.0003%), with 10 alterations never previously reported. In addition, a number of non-rare variants in genes relevant to cancer risk (e.g. in BRCA1, PALB2, BLM, and others) were detected in a significant number of individuals.

**Table 3:** Selected variants with scores of amino acid damage from 5 predictors and variant frequency in ExAC, by patient

Patient ID	Variant DNA level	Gene	Consequence	Non-neutral scores	Representation in ExAC (European non-Finnish)	
					Allele count	Allele number
**112940**	9:32989766 G/A	APTX	NP_001182178.1 p.R56X	5†	**0**	66736
	17:41246481 T/C	BRCA1	NP_009225.1 p.Q356R	4	**4198**	66734
	4:178274801 T/G	NEIL3	NP_060718.2 p.F460C	3	**10**	66730
**117197**	22:43933284 CCT/C	EFCAB6	NP_073622.2 p.Q1340Rfs*43	5†	**606**	66684
	2:38301879 T/A	CYP1B1	NP_000095.2 p.D218V	5	**15**	41314
	10:89503283 C/T	PAPSS2	NP_004661.2 p.P454L	5	**0**	66732
	17:41246481 T/C	BRCA1	NP_009225.1 p.Q356R	4	**4198**	66734
	9:135779052 G/A	TSC1	NP_000359.1 p.H732Y	4	**350**	66706
**117939**	1:156212872 T/A	BGLAP	NP_954642.1 p.C74X	5†	**8**	66696
	2:58386928 G/GTAAT	FANCL	NP_060532.2 p.T367Nfs*13	5†	**232**	65648
	5:80109533 T/C	MSH3	NP_002430.2 p.I929T	5	**0**	66740
	12:124209215 G > T	ATP6V0A2	NP_036595.2 p.K103N	5	**15**	66734
	3:51673972 A/T	RAD54L2	NP_055921.2 p.I730F	4	**−**	−
	21:16340242 T/C	NRIP1	NP_003480.2 p.E91G	4	**−**	−
	2:149226489 C/T	MBD5	NP_060798.2 p.A326V	3	**−**	−
**123136**	4:1206089 G/A	CTBP1	NP_001319.1 p.421L	4	**28**	14670
	3:38888684 A/T	SCN11A	NP_054858.2 p.F1626Y	3	**−**	−
	1:63876815 A/G	ALG6	Splice acceptor (−2)	−	**−**	−
	1:120056817 T/TGCA	HSD3B1	NP_000853.1 p.V224_Y225insH	−	**2**	66708
	4:153332604 TCTC/T	FBXW7	NP_361014.1 p.E117del	−	**35**	66114
**124604**	16:23634293 C/T	PALB2	NP_078951.2 p.G998E	5	**1430**	66736
	16:89815152 G/A	FANCA	NP_000126.2 p.S1088F	4	**4798**	65430
	15:91326099 C/T	BLM	NP_000048.1 p.P868L	4	**4239**	66162
	6:49700908 G/A	CRISP3	NP_006052.1 p.A197V**	2	**0**	66362
**124853**	18:3452067 G/A	TGIF1	NP_733796.2 p.W30X	5†	**33**	66002
	4:55955969 C/T	KDR	NP_002244.1 p.A1065T**	5	**52**	66726
	17:12901781 A/C	ELAC2	NP_060597.4 p.S490A	5	**39**	66734
	19:50766628 C/T	MYH14	NP_001139281.1 p.A882V	3	**23**	27644
	X:110973633 TGAA/T	ALG13	NP_001093392.1 p.E795del	−	**33**	41558
	4:103747794 C/T	UBE2D3	Splice acceptor (−1)	−	**−**	−
**125671**	9:35707745 G/C	TLN1	NP_006280.3 p.L1539V	4	**13**	66734
	1:145578236 C/T	PIAS3	NP_006090.2 p.R67W	3	**13**	66740
	10:5014483 T/A	AKR1C1	NP_001344.2 p.S221N	3	**119**	66712
	10:5014484 C/A	AKR1C1			**119**	66712
	11:47237894 CAGA/C	DDB2	NP_000098.1 p.R47del	−	**−**	−
**126002**	17:35564593 G/A	ACACA	NP_942134.1 p.R1182W	5	**16**	66612
	17:41246481 T/C	BRCA1	NP_009225.1 p.Q356R	4	**4198**	66734
	7:18633593 A/G	HDAC9	NP_001191074.1 p.Y199C	3	**0**	66702
**129413**	14:50088465 T/G	MGAT2	NP_002399.1 p.I160S	5	**610**	66402
	17:41246481 T/C	BRCA1	NP_009225.1 p.Q356R	4	**4198**	66734
	15:43762077 TGGGATA/T	TP53BP1	NP_001135451.1 p.I455_P456del	−	**−**	−
**129547**	2:38298287 T/TGGTGGCATCA	CYP1B1	NP_000095.2 p.T404Sfs*30	5†	***	
	10:94297192 C/T	IDE	NP_004960.2 p.G72S	5	**8**	66724
	12:124824917 C/T	NCOR2	NP_001070729.2 p.R1794Q	3	**2**	65378
	21:16337279 C/A	NRIP1	NP_003480.2 p.V1079F	3	**44**	66670
**129748**	16:23632788 TTTTC/T	PALB2	NP_078951.2 p.E1002Tfs*4	5†	**−**	−
	6:52657698 C/T	GSTA1	NP_665683.1 p.E168K	4	**1**	66738
	8:48973252 G/A	UBE2V2	NP_003341.1 p.R101Q	4	**1**	65850
	20:31021718 C/T	ASXL1	NP_056153.2 p.R573W**	4	**4**	63434
	12:53776449 G/C	SP1	NP_612482.2 p.G240R	3	**26**	66738
	11:62388048 G/C	B3GAT3	NP_036332.2 p.R60G	3	**1**	60290
**131534**	9:131709581 A/AT	DOLK	NP_055723.1 p.M1?	5†	**823**	63350
	13:28592620 T/C	FLT3	NP_004110.2.Y842C	5	**2**	66710
	10:94274700 A/G	IDE	NP_004960.2 p.M254T	5	**11**	66698
	4:70723282 C/G	SULT1E1	NP_005411.1 p.W27C	4	**−**	−
	1:120478125 A/C	NOTCH2	NP_001186930.1 p.F1209V	4	**306**	66726
	17:33430313 T/C	RAD51D	NP_001136043.1 p.E223G	4	**874**	51128
	1:182555767 C/T	RNASEL	NP_066956.1 p.G59S	4	**379**	66514

Nicolas et al., Table 3

5† score given to variant creating stop gain or frameshift;

** variant causing missense and located in splice site;

*** this variant has been described in patients with congenital glaucoma, an autosomal recessive trait usually recognized during the first year of life (Sena, et al., 2004). The mutation is not listed in ExAC as it excludes mutations associated with severe pediatric diseases. In the representation in the ExAC column, (−) denotes that the variant was not found in the database while (0) denotes that the variant was absent in European non-Finnish but detected in other ethnicities, as listed in Supp Table 4. This Table also lists damaging variants in genes shown to be clearly tumor-promoting in some inherited or somatic forms of other cancers, although not currently well validated for prostate, including FLT3, ASXL1, KDR, NOTCH2 (e.g. (Kindler, et al., 2005; Sallmyr, et al., 2008; Antonescu, et al., 2009)); as well as genes which are identified by the candidate criteria noted in Table 2, but for which limited information is available based on functional characterization to date (e.g., the AR-interacting protein IDE interacts directly with AR (Kupfer, et al., 1994)).

### High frequency of variants affecting genes involved in DNA repair and androgen signaling

19 variants affecting the function of genes involved in DDR (with some known to be androgen regulated), and AR-dependent transcription were identified in the patient cohort, with 11/12 patients having at least 1 affected gene in this category, and 5/12 having 2 or more. These genes included PALB2 (also known as FANCN), *APTX, BLM, BRCA1, CTBP1, DDB2, FANCA, FANCL, MBD5, MSH3, NEIL3, RAD51D, RAD54L2* (also known as ARIP4), *SP1, TP53BP1, UBE2D3, UBE2V2* (also known as MMS2). Many of the proteins encoded by these genes interact to mediate DNA repair functions (Figure 2B, 2C)

For some patients, a single variant seemed likely to pose substantial risk, such as the frameshift variant E1002Tfs*4 in *PALB2* found patient 129748. This patient was diagnosed at age 41, with a father who was also diagnosed with prostate cancer at age 67. PALB2 truncating mutations have been detected in patients with Fanconi's anemia and various cancers [18], including prostate [15]. Importantly, PALB2 p.E1002Tfs*4 lacks part of the WD40 repeat domain (amino acids 853–1186) known to mediate protein interactions with key proteins involved in homologous recombination (HR) such as BRCA2 and RAD51 [42, 43]. Potentially magnifying the effect of the PALB2 truncation, this patient also has a mutation (p.R101Q) predicted to damage in the ubiquitin conjugating enzyme UBE2V2, identified as a factor required for avoidance of UV damage [44], with expression of UBE2V2 linked to prognosis in breast cancers following treatment with DNA damaging therapies [45].

Some patients had suggestive combinations of rare variants. For example, patient 117939 has three independent mutations likely to directly impact DNA damage response: FANCL p.T367Nfs*13, MSH3 p.I929T, and RAD54L2 p.I730F (Figure 2B). FANCL is an E3 ubiquitin ligase of the Fanconi Anemia (FA) core complex. The mutation T367Nfs*13 produces a protein with premature truncation and three novel amino acids at the C-terminus, and has been described in an FA patient [46]. This mutation produces a hypomorphic mutation with only partial correction of mitomycin C sensitivity and chromosomal defects [46]. MSH3 heterodimerizes with MSH2 to mediate mismatch repair; variants in MSH3 have been associated with risk of some forms of cancer, including prostate [47, 48]. Importantly, crosstalk between FA and MSH2/MSH3 in the mismatch repair pathway has been well documented, with the two operating as redundant DNA damage sensors [49–51]. RAD54L2/ARIP4 is a DNA helicase that binds the AR, and modulates AR-dependent transactivation in a promoter-dependent manner, and has been linked to a role in DNA repair [48, 52, 53]. I730 is just downstream of one of the three LXXLL motifs, also known as nuclear boxes, that mediate protein-protein interactions.

In some cases, combinations involving non-rare variants of DDR genes were suggestive. Patient 124604, diagnosed with prostate cancer at age 43, has a pedigree that shows cases of prostate cancer over 3 generations. This patient carries 3 non-rare missense variants in genes involved in DDR: BLM p.P868L, PALB2 p.G998E, and FANCA p.S1088F. *BLM* encodes a 3′-5′ DNA helicase which functions in maintenance of genomic stability, with inactivating mutations associated with a progeria, Bloom Syndrome (BS). BLM p.P868L has been characterized as an allele that is unlikely to cause BS, but causes partial loss of function manifested by an intermediate sensitivity to hydroxyurea [54], and has been associated with increased rectal cancer risk [55]. Interestingly, an uncle of patient 124604 had colorectal cancer. The PALB2 p.G998E variant in this patient was reported at a similar frequency of ~ 10% in a population of BRCA1- and BRCA2-negative male breast cancer patients in Northern Italy as was observed in healthy individuals [56]. A similar high rate of occurrence in normal populations was observed for FANCA p.S1088F (9/97 in breast cancer cases *vs* 11/94 in controls) [57]. However, the assortment of three independent alleles affecting DNA repair in the pedigree of this patient may have an additive effect, given the involvement of all three proteins in related DNA repair pathways (Figure 2B).

In a similar case, Patient 112940 had a rare variant causing a stop at amino acid 56, eliminating function of APTX (aprataxin), involved in the repair of multiple forms of DNA breaks and implicated in therapeutic response in cancer [58–60], and a second rare variant (p.F460C) damaging NEIL3, a DNA glycosylase involved in the base excision repair pathways that protects cells from genotoxic stress and has been associated with prostate cancer risk [61, 62]. Interestingly, this patient as well as 3 other unrelated individuals (Patients 117197, 126002, and 129413) all had the same non-rare variant in BRCA1, p.Q356R, which some prior studies have linked to prostate cancer risk [63]; an observed incidence of 33%, versus the expected incidence of this variant should be 9%, based on ExAC. Like patient 112940, the other patients also possessed multiple additional candidate rare variants affecting DNA damage response and/or genes related to androgen function. Patient 129413 had a mutation disrupting TP53BP1 (p.I455_P456del, predicted to be deleterious by PROVEAN), TP53BP1 competes with BRCA1 for directing proteins down the non-homologous end joining (NHEJ) versus homologous recombination (HR) repair pathway (Figure 2C) [64–67]; in the context of impaired TP53BP1, or other DDR defects, and as discussed further below, the BRCA1 variant may have a more deleterious effect.

**Figure 3:**
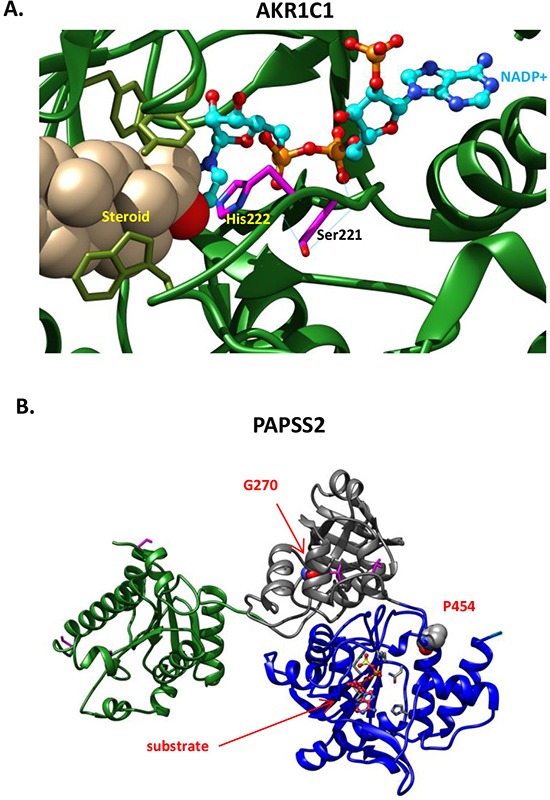
Functional defects associated with novel missense variants **A.** AKR1C1, S221N. AKR1C1 catalyzes the inactivation of progesterone to the less potent 20α-hydroxyl-pregn-4-ene-3-one. The reaction is NADPH dependent with an obligatory requirement for the cofactor to bind before the steroid substrate can bind to form the central complex. The progesterone is maintained in a steroid binding site at H222; an H222I mutation decreases the Km value for NADPH 95-fold [102]. Here, AKR1C1 (PDB code: 1MRQ) is shown with bound steroid 20alpha-hydroxy-progesterone, and the cofactor, NADP+ in ball-and-stick representation with cyan carbons, and orange phosphorus atoms. S221 and adjacent catalytic residue H222 of AKR1C1 are shown with magenta sticks. S221 is involved in 2 hydrogen bonds (shown with cyan thin lines) with adjacent residues and one with the NADP+ cofactor. Though predicted to be benign by several conservation based servers, the S221N substitution disrupts the hydrogen-bonding network required to maintain the catalytic active site configuration. **B.** Shown are the PAPSS2 kinase domain in green, PUA (PseudoUridine synthase and Archaeosine transglycosylase) domain in gray and sulfate adenylyltransferase domain in blue. The position of the P454L and G270D missense variants are indicated.

In some cases, patients have mutational profiles in which disruption of DDR and AR signaling is closely linked. For example, patient 125671 has an in-frame deletion (p.R47del) in the AR-interacting protein DDB2: the R47 residue mediates high affinity binding of DDB2 to damaged DNA [68, 69]. This patient also has an S221N variant predicted to be highly deleterious (Figure 3A) in AKR1C1, a member of an enzyme family that controls concentrations of active androgens and other steroidal hormones, regulates *trans*-activation of AR in the prostate, and has been shown to regulate resistance to the anti-androgen enzalutamide, recently approved for treatment of castration resistant prostate cancer [70, 71].

Patient 123136 comes from a family with a high burden of cancer, with a sister affected with melanoma and breast cancer, father with lung cancer, and mother with colon cancer. This patient has a rare variant (p.P421L) of CtBP1, a coregulator of BRCA1, which has been linked to risk of prostate cancer [72, 73]. Activation of the AR regulator HIPK2 (Homeodomain-Interacting Protein Kinase 2) [74] by genotoxic stress triggers apoptosis in part through phosphorylation of CtBP1, which causes CtBP1 degradation [75]; loss of this signaling could plausibly cause predisposition to multiple forms of cancer. P421L destroys the HIPK2 phosphorylation site on CtBP1.

### Other classes of variants

In addition to the selected examples described above, rare variants were identified in all of the categories of interest from the candidate list. Due to space limitations, all profiles cannot be fully detailed. However, some observed allelic combinations are of interest, particularly in regard to androgen availability and AR-dependent transcription. Consideration of these variant combinations may be particularly important in suggesting a possible explanation for why mutations in the DDR machinery, which theoretically could increase risk of any type of cancer, could result in a familial predilection for prostate cancer.

As selected examples, two patients, 129547 and 117939, had disruptive mutations in NRIP1 (also known as RIP140), a co-receptor for estrogen, androgen, and other classes of nuclear hormone receptor. Disruption of NRIP1 function has been reported to lead to hyperactivation of AR signaling [76], and variants in NRIP1 have been linked to risk in breast, endometrial, and other cancers [77, 78]. Interestingly, patient 129547 also has a rare variant affecting a second AR co-repressor, NCOR2 (also known as SMRT) that limits AR signaling [79], while patient 117939 has a rare variant in an alternative AR cofactor, RAD54L2 (also known as androgen receptor interacting protein 4, ARIP4) [80]. These variant pairs may interact to de-repress AR signaling in these two patients. Another patient, 117197, has a frameshift variant (Q1340Rfs*43) in EFCAB6 (also known as DJBP), which encodes a protein that recruits histone-deacetylase (HDAC) complexes to repress AR-dependent transcription [81]; the variant eliminates the HDAC-interaction domain. Patient 124604, noted above as having 3 variants in DDR-related proteins (BLM, PALB2, and FANCA), has a family with prostate cancer over 3 generations. This patient also has a splice site-disrupting variant in CRISP3 (cysteine-rich secretary protein 3). Expression of CRISP3 is prostate-specific, and CRISP3 is up-regulated in a subset of prostate cancers [82], especially prostate cancer with the TMPRSS2-ERG fusion gene [83]. Mis-splicing due to the G/A mutation in 6:49700908 would destroy the CRISP domain (pfam: 08562), which allows CRISP3 to regulate ryanodine receptor Ca2+ signaling. Interestingly, ExAC data indicates the position is multi-allelic with another allele (T) mainly represented in African populations.

A major function of the prostate is in production of glycoproteins, including PSA, that support sperm production, and changes in glycosylation are associated with prostatic neoplasms [84] and response to androgen treatment [85]. Among a number of variants in genes linked to glycosylation defects (PAPSS2, ATP6V0A2, ALG13, MGAT2, B3GAT3, DOLK), the variant in PAPSS2 seems the most interesting. This variant (p.P454L; Figure 3B) is strongly predicted to destroy the catalytic function of PAPSS2, a kinase and ATP sulfurylase that catalyzes two sequential reactions to synthesize PAPS, the sulfate source for sulfation of the androgen precursor didehydroepiandrosterone (DHEA). Of particular relevance to prostate cancer, two mutations in PAPSS2 have been reported as causing androgen excess via complete (W362Cfs*3) or partial (G270D) disruption of DHEA sulfation [86].

### Functional defects in response to DNA damaging agents in the lymphocytes of patients of prostate cancer patients with familial risk

**Figure 4:**
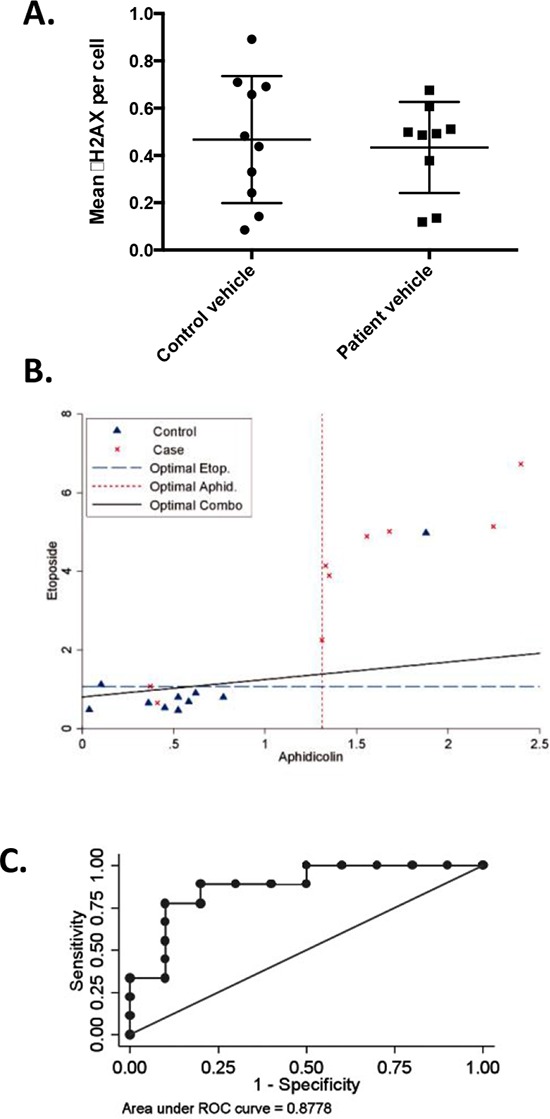
Elevated γH2AX in T-cells from patients with prostate cancer following treatment with DNA damaging agents Primary T-cells from 9 patients and 10 age- and sex-matched controls were stimulated by PHA and IL-2, then treated with vehicle, aphidicolin or etoposide, and stained for nuclear γH2AX foci. **A.** Mean number of γH2AX foci in vehicle treated patients and matched controls (*p* = 0.746, not significant). **B.** Mean number γH2AX foci per cell are depicted for cases and controls following drug treatment. Cases: red ‘x's, controls: blue triangles. Dashed lines: statistically optimal cutoff points to discriminate between samples with high and low γH2AX levels for each treatment or the two tests combined, as indicated. Using the combination (solid black line), 7/9 patients exhibited high γH2AX levels versus 1/10 controls. **C.** Area under the receiver operating characteristic (ROC) curve (AUC) for the combined γH2AX scores for aphidicolin and etoposide demonstrates assays discriminate between patients and controls, AUC = 0.8778.

Given the strong implication of defects in genes involved in DNA repair as causative factors for numerous classes of hereditary cancer, these genes were of particular interest. PBLs were available for 9/12 patients, allowing us to assess whether the response of these cells to low doses of DNA damaging agents differed from those of 10 age and gender-matched individuals without a cancer diagnosis or a family history of cancer. Immunofluorescence was used to assay the formation of DSB-associated γ (phospho)-H2AX foci in cells with and without treatment with the DNA polymerase inhibitor aphidicolin or the topoisomerase II inhibitor etoposide. Under baseline conditions without drug treatment, the patient and control groups were statistically indistinguishable (*p* = 0.746) (Figure 4A). However, treatment of cells with low doses of aphidicolin (*p* = 0.0337) or etoposide (*p* = 0.007) revealed significant differences in γH2AX induction between cases and controls, with a higher magnitude of induction seen in prostate cancer patients (Figure 4B). Receiver operating characteristic (ROC) curve analysis for the combination of both treatments indicated specificity and selectivity of observed differences were 87% (Figure 4C).

## DISCUSSION

With the growing availability of powerful technologies for genomic analysis, cancer risk assessment is at a key point of potentially benefitting from advances in sequencing. Some large families with detailed family cancer history are in the unambiguous situation of having a well-defined mutation in a highly penetrant gene. However, a significant proportion of families have limitations in family cancer history information, making exploratory studies such as this one useful in defining strategies for potential clinical evaluation. In this study, we have focused most effort on the analysis of defects in DDR in case-only individuals with prostate cancer and strong family cancer history. This approach has identified variants or variant combinations in almost every patient assessed that have the potential to alter response to DNA damage, with findings further supported by direct demonstration of elevated expression of γH2AX following administration of DNA damaging stimuli. In the group of patients we examined, variants often occurred in combinations that would be predicted to interact to weaken DDR, and typically each variant was unique to the individuals investigated. These findings suggest that DDR defects may play a role in prostate cancer predisposition, similar to other hereditary cancers such as breast [87] and colorectal [88]. Two recent studies identified a high burden of germline mutations affecting DDR genes in somatic prostate cancers [89–91].

This study also suggests that non-rare variants affecting DDR may play a role in prostate cancer risk upon further study. For example, in the cohort analyzed here, 33% of patients had the same non-rare p.Q356R variant in BRCA1. BRCA1 p.Q356R is an example of a variant that has a frequency of minor allele over 5% and an increased odds ratio for breast cancer over 1.5 in carriers. In a study on association between prostate cancer risk and SNPs in a 200 kb area around the BRCA1 gene, the strongest link was for BRCA1 p.Q356R [63], with the R allele preferentially transmitted to men affected with prostate cancer before the age of 50. Mechanistically, Q356 is of particular interest as it is located in the MRE11/RAD50/NBS1 (MRN) domain, required for interaction with RAD51, p53, ZBRK1, SWI/SNF, BRAP2, ATPase, and Importin α [92]. Q356R disrupts the interaction between BRCA1 and ZBRK1, eliminating the transcriptional co-repressor function of BRCA1 [93], leading to upregulation of such BRCA1 target genes as angiopoietin-1 (ANG1), which promotes angiogenesis and proliferation [94]. In a study of 931 PCa patients, 13 independent variants of uncertain significance were identified in the MRE11/RAD50/NBS1 domain including Q356R [15]. Our data raises the idea that BRCA1 Q356R may be significant in the context of other variants that weaken DDR.

DDR genes are active in many tissues; hence, the fact that the patients analyzed developed prostate cancer as well as multiple other cancers in their families may reflect the contribution of multiple modifier variants. Our candidate approach also considered variants predicted to influence signaling by androgens and other hormones, or prostate-specific functions such as control of glycosylation and a number of damaging rare variants were also found in these genes in the sequenced probands. Our data suggests these gene pathways are worth following as genomic characterization of prostate and other hereditary tumors advances.

An important issue is how to determine if detected germline variants are indeed functional to inform cancer risk assessment. Recent studies have reported that evaluations of the germline DNA of “normal” individuals identifies a surprisingly high burden of variants that would seem to be predisposing to cancer and other diseases [11, 95]. Furthermore, variants may be detected in genes that are not expressed in the prostate or affected tissue [96–98]. These points highlight the need for functional studies to follow-up on detection of germline variants. In this study, the functional testing of patient-derived PBLs has supported the idea that responses to DNA damage in prostate cancer patients differ from those found in age and gender-matched controls. This approach can potentially be extended using systematic cell-based functional assays for phenotyping of missense alleles [99].

Ultimately, these results suggest that future strategies for risk assessment may involve a diagnostic algorithm in which high-risk patients initially are tested for well-validated, high penetrance variants, using standard panel-based screens. If initial testing fails to identify causative mutations, exome-testing focusing on cancer-relevant processes, supported by functional testing for defects in the process, may be a useful alternative approach that should be evaluated in a prospective setting. Another potentially valuable aspect of such broader testing may be implications for patient treatment. Identifying a variant that renders the cell dependent on a specific pathway may create an opportunity for synthetic lethality, as in the example of BRCA mutations and PARP inhibitors [100]. Broader approaches for functional assessment of germline variants are also needed to better inform cancer risk assessment and recommendations.

## MATERIALS AND METHODS

**Figure 1:**
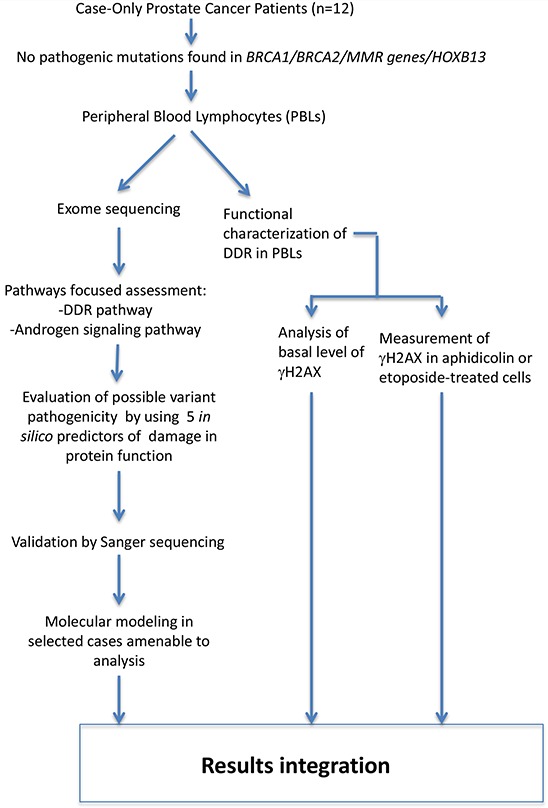
Project Flow Chart After review of family history and negative results for genetic testing for known predisposing factors, 12 DNA samples isolated from peripheral blood lymphocytes (PBLs) of 12 patients with prostate cancer were sent to Ambry Genetics for exome sequencing. Data analysis included identification of the rare variants in 826 genes selected as described in Table 2 and validation by Sanger sequencing of the variants that scored non neutral by at least 3 *in silico* predictors. Two variants (in AKR1C1 and PAPSS2) were further characterized by molecular modeling. PBLs were also used to assess the response to DNA damaging agents.

A flowchart for the analysis in the study is provided in Figure 1.

### Patient selection

Case-only prostate cancer patients included in this study (*n* = 12) had undergone evaluation for inherited cancer risk. Each participant had a strong family cancer history as shown in Table 1, with either multiple first-degree or second-degree relatives with prostate or other cancers. The mean age at prostate cancer diagnosis was 57.8 years (range 41–68 years). Fifty-eight percent had Gleason score > = 7, and 25% had advanced stage disease (T3). Hereditary cancer syndromes evaluated in these families included hereditary breast-ovarian cancer (HBOC), Lynch syndrome (LS), and hereditary prostate cancer (HPC) [22–24]. No pathogenic mutations were identified from sequencing the following genes: *BRCA1, BRCA2, MLH1, MSH2, PMS2, MSH6*, and *HOXB13*. These patients had consented to the FCCC Risk Assessment Program Registry, which allowed further research genomic sequencing. All patients reported being white, non-Hispanic. Peripheral blood DNA from these 12 prostate cancer patients was sent to Ambry Genetics for exome sequencing.

**Table 1:** Family history and prostate cancer characteristics of analyzed prostate cancer patients

Patient ID	Age at diagnosis	Stage/Gleason	1^st^ degree relatives with cancer Type of cancer/age	2^nd^ degree relatives with cancer Type of cancer/age
**112940**	68	T2aN0MX/3+4=7	sister-ovary 27 and colon 66 sister-abdominal cancer early 60′s brother-prostate 59 mother-brain 52 father-unknown cancer	
**117197**	68	T3bN0MX/4+3=7	mother-stomach 80, colon 95	nephew-non Hodgkin's 20′s maternal uncle-unknown cancer maternal grandmother-colon 69 paternal uncle-prostate 62 paternal uncle-mouth 88
**117939**	65	T2cNOMX/3+3=6	father-prostate 65 2 siblings w polyps	paternal uncle-throat 60 paternal uncle-prostate 65 maternal grandmother-breast 30 nephew-small cell desmoplastic tumor 28
**123136**	59	T2cNXMX/6	mother-colon mid 40′s father-lung 59 daughter-melanoma 25	
**124604**	44	T2cN0MX/3+3=6	father-melanoma 72, polyps	paternal uncle-colon 50 maternal uncle-prostate cancer 62 maternal uncle-prostate cancer 55 maternal grandfather-prostate cancer 88
**124853**	65	T2cNOMX/3+3=6	sister-basal cell 60 mother-stomach 59	paternal aunt-breast paternal cousin's daughter-ovarian 50′s
**125671**	54	T2cN0MX/3+3=6	sister-uterine 54 sister-non Hodgkin's 37 father-prostate 72 mother-kidney 76	paternal cousin-breast 36 paternal cousin-colon 58 maternal uncle-unknown cancer 30
**126002**	59	T3aN0MX/3+4=7 and 4+3=7 and 6 (multiple areas)		maternal aunt-unknown cancer 89 maternal cousin - colon 65 maternal cousin-brain 50′s maternal 1st cousin-leukemia 7-breast/skin 40′s paternal 1st cousin-brain 59
**129413**	57	T1c/3+4=7	father-prostate 70, kidney 80, liver 80 sister-precancerous uterine 47	paternal grandfather-prostate 85 paternal great-grandfather-stomach paternal uncle-prostate 66
**129547**	62	T2cN0MX/3+4=7	sister-ovary 57, cervix 57	paternal cousin-inflammatory breast 45 maternal uncle-prostate 78 maternal uncle-prostate 78 maternal 1/2 uncle-pancreas 56 maternal 1/2 uncle-prostate 78 2 maternal cousins-unknown cancer
**129748**	41	T3bN1M0/4+4=8	father-prostate 67	paternal grandfather-polyps paternal great aunt-breast 30′s
**131534**	52	T2cNO/3+4=7	mother-breast 68, melanoma 65 father-prostate 70	maternal aunt-colon 45 maternal aunt-polyps 40 maternal cousin-glioblastoma 26 paternal grandmother-lung

### Exome sequencing

Exome sequencing of germline DNA was performed by Ambry Genetics (Aliso Viejo, CA) at 30X average coverage using a VCRome kit (Roche Nimblegen, Madison WI) for library preparation, indexing and 100 bp paired end processing using the HiSeq platform (Illumina, Hayward, CA). Human hg19 reference-guided alignment and variant calling were done using the Illumina CASAVA software pipeline. Heuristic filtering processes were applied to remove variants that fall into non-coding regions, with synonymous effect, or common variants found in the 1000 genomes, dbSNP or Exome Sequencing Project (ESP) database. The Ambry Variant Analyzer (AVA™) produced candidate mutation short lists of rare variants by restricting to variants fitting a dominant/recessive model of inheritance, as well as listing variants associated with hereditary and somatic cancers, regardless of frequency (i.e., including non-rare variants).

### Development of a high value list of candidate genes

The candidate gene list (Supp Table S1) was assembled from the sources listed in Table 2. Genes collected from various sources were prescreened for possible aliases by using G-convert from G-profiler (http://biit.cs.ut.ee/gprofiler/gconvert.cgi) in batch mode to ensure use of official gene symbols with Entrez ID numbers. Manual curation using (http://www.genenames.org/) from the Human Genome Organization (HUGO) Gene Nomenclature Committee (HGNC) was done when G-convert did not return information. PCAP, PCA3 and HPC6, which were listed as loci relevant to prostate cancer by at least one source, were not included as they fall under the HGNC locus type “phenotype only”, indicating the causative gene has not been identified. Non-coding transcripts such as PCAT4, 5 and 6 and pseudogenes were excluded.

### Variant selection

By analysis through its AVA™ filtering software, Ambry produced lists of rare variants (defined as frequency less than 1% in the general population) for each patient. From these combined lists, 84 single nucleotide variations (SNVs) with more than 3 reads, Q score above 25 and leading to non-synonymous changes at the protein level in candidate genes were extracted. Variants leading to non-synonymous changes in encoded proteins were selected if they received scores indicating a protein-damaging function with at least 3 of 5 *in silico* predictors (PolyPhen-2 with HumDiv as model classifier [25], SIFT [26], PROVEAN [27], MutationAssessor [28], and MutationTaster [29]). The conversion of the calls made by each predictor into neutral *vs*. non-neutral was made using an approach that integrates different predictive algorithms [12]. For PolyPhen-2, “probably damaging” and “possibly damaging” were considered non-neutral. For MutationAssessor, “high” and “medium” were considered non-neutral. For MutationTaster, “disease causing” and “disease causing_automatic” were considered non-neutral and “polymorphism” and “polymorphism_automatic” neutral. “Damaging” (SIFT) and “deleterious” (PROVEAN) were considered non-neutral while “tolerated” (SIFT) and “neutral” (PROVEAN) were considered neutral. For MutationTaster, 0.99 was used as cut-off in the disease_causing category.

In-frame deletion (Indel) variants were characterized with PROVEAN and MutationTaster. Indels that had a length divisible by 3 and caused amino acid insertion/deletion (also called 3N indels) were also analyzed with SIFT-Indel (http://sift-dna.org) [30]. Variants associated with possible splicing defects were also selected. Mutalyzer 2.0.4 (http://mutalyzer.nl/) was used to ascertain that the variants were described according to the Human Genome Variation Society (HGVS) nomenclature, effectively matching described amino acid and nucleotide coordinates to the GRCh37/hg19 assembly [31].

### Variant verification

The Exome Aggregation Consortium (ExAC) website, Cambridge, MA (version 0.3) (http://exac.broadinstitute.org/) was used to assess the frequency of the selected variants in the general population or in a particular ethnic group. The ExAC data set contains information on 60,706 unrelated individuals sequenced as part of various disease-specific and population genetic studies, approximately 50% of who are of European non-Finnish ancestry. A second independent group of controls, referred to as ITMI genomes and representing an extension of the set initially described in [11], consisted of 634 white non-Hispanic individuals who denied a personal or family history of cancer. For all variants with predicted protein-damaging consequences, primers flanking the variation were designed to amplify a product of ~200 to 400 base pairs. After digestion with ExoSap-IT (Affymetrix, Santa Clara, CA), the PCR product was sent to Genewiz (South Plainfield, NJ) for Sanger sequencing.

### Molecular modeling

For analysis of structural consequences of missense variants, models of PAPSS2 and AKR1C1 were generated. All molecular display figures were prepared with the UCSF Chimera software [32]. While the N-terminal kinase domain is of known structure (2AX4), a full-length model of PAPSS2 was generated with Biological Assembly Modeler [33] based on the closest homolog of known structure, PAPSS1 (PDB code 1XNJ, 77% identity, 87% similarity), and compared with a previously deposited model [34]. An alternate template structure (2QJF) was superposed to extract the placement of the substrate and product molecules, (ADP and Adenosine-5′-phosphosulfate). For the variant in AKR1C1, the structure with PDB code 1MRQ was used.

### Lymphocyte cell preservation, culture, and analysis of DNA damage response (DDR)

Peripheral blood lymphocytes (PBLs) were available from 9/12 of the exome sequenced patients and 10 age-matched and gender-matched individuals without a cancer diagnosis or a family history of cancer. Control samples were obtained from the FCCC Biosample Repository Facility. For analysis of DDR, cells were cultured in RPMI-1640 containing 15% fetal bovine serum (HyClone Laboratories, Logan, UT), 2 mM L-glutamine (Life Technologies, Grand Island, NY), 50 μM 2-mercaptoethanol (Sigma-Aldrich, St. Louis, MO), 0.2 units human recombinant insulin (Sigma) per ml, 50 units penicillin and 50 mg streptomycin per ml (complete RPMI), and then stimulated with phytohemagglutinin (PHA)-M (Life Technologies, Grand Island, NY) and recombinant human interleukin 2 (IL-2) (NCI Preclinical Repository) for 72 hours. Cells were then treated with vehicle, 10 μM aphidicolin or 25 μM etoposide, and fixed in paraformaldehyde 2 hours later. For immunofluorescence, cells were allowed to attach to poly-d-lysine-coated 96-well plates, stained with anti-γH2AX antibody (#05–636, Millipore, Temecula, CA). Sixteen images per well were acquired at 20X (with each image acquired in 2 channels to detect γH2AX with TRITC and total DNA with DAPI) utilizing the ImageXpress micro automated microscope (Molecular Devices, Sunnyvale, CA) driven by MetaXpress software. Images were analyzed in the Multiwavelength Scoring module of MetaXpress and results were displayed and exported utilizing the AcuityXpress software package (Molecular Devices, Sunnyvale, CA).

## SUPPLEMENTARY TABLES










