# Supplementary material for: Systematic evaluation of underlying defects in DNA repair as an approach to case-only assessment of familial prostate cancer
Source: Oncotarget. 2015 Oct 14;6(37):39614–33. doi: 10.18632/oncotarget.5554 (PMC4741850; doi:10.18632/oncotarget.5554)
Supplement: Supplementary file 1 [file oncotarget-06-39614-s001.pdf]

SUPPLEMENTARY TABLES

Supplementary Table S1: Candidate genes list with sources nominating the genes. The 826 candidate gene list was assembled from the sources listed in Table 2. This identifies the source(s) of nomination for each gene. A ranking number is indicated for the 3 lists that were ranked.

Supplementary Table S2: *In silico* predictor scores for single nucleotide variants (SNVs) detected in the 12 patients. All SNVs with more than 3 reads, Q scores higher than 25 and passing the candidate gene list filter are scored with 5 *in silico* predictors. Variants that scored non-neutral by at least 3 predictors (as defined in the Material and Method section) are considered for Sanger validation.

Supplementary Table S3: Small in-frame deletions (Indels). Provean, MutationTaster and SIFT-Indel entries for the Indel variants listed in Table 3, showing a damaging score by at least one predictor for each of the selected variants.

Supplementary Table S4: Full variant description and representation in various populations. In this Supplement to Table 3, the data presented in Table 3 are in bold. Additional information includes variant identification by rs number (when available), description at the cDNA level, and representation in the general population and in a set of white non-Hispanic without history of cancer in family (ITMI genomes set) (Bodian, et al., 2014).
